# The prevalence of non-pharmacological interventions in older homecare recipients: an overview from six European countries

**DOI:** 10.1007/s41999-023-00868-w

**Published:** 2023-10-04

**Authors:** Eline C. M. Kooijmans, Emiel O. Hoogendijk, Jitka Pokladníková, Louk Smalbil, Katarzyna Szczerbińska, Ilona Barańska, Adrianna Ziuziakowska, Daniela Fialová, Graziano Onder, Anja Declercq, Harriet Finne-Soveri, Mark Hoogendoorn, Hein P. J. van Hout, Karlijn J. Joling

**Affiliations:** 1 Department of General Practice, Amsterdam UMC, Location Vrije Universiteit Amsterdam, De Boelelaan 1117, Amsterdam, The Netherlands; 2 Amsterdam Public Health, Ageing and Later Life, Amsterdam, The Netherlands; 3 Department of Epidemiology and Data Science, Amsterdam UMC, Location Vrije Universiteit Amsterdam, De Boelelaan 1117, Amsterdam, The Netherlands; 4 Department of Social and Clinical Pharmacy, Faculty of Pharmacy in Hradec Králové, Charles University, Prague, Czech Republic; 5 Department of Computer Science, Vrije Universiteit Amsterdam, De Boelelaan 1111, 1081 HV Amsterdam, The Netherlands; 6 Chair of Epidemiology and Preventive Medicine, Laboratory for Research on Aging Society, Medical Faculty, Jagiellonian University Medical College, ul. Skawińska 8, Kraków, Poland; 7 Department of Geriatrics and Gerontology, 1st Faculty of Medicine in Prague, Charles University, Prague, Czech Republic; 8 Fondazione Policlinico Gemelli IRCCS and Università Cattolica del Sacro Cuore, Rome, Italy; 9 LUCAS-Center for Care Research and Consultancy and Ceso-Center for Sociological Research, KU Leuven, Leuven, Belgium; 10 Finnish Institute for Health and Welfare, Helsinki, Finland; 11 Department of Medicine for Older People, Amsterdam UMC, Location Vrije Universiteit Amsterdam, De Boelelaan 1117, Amsterdam, The Netherlands

**Keywords:** Older people, Homecare, Non-pharmacological interventions, Care for older persons, Non-drug interventions

## Abstract

**Purpose:**

Non-pharmacological interventions (NPIs) play an important role in the management of older people receiving homecare. However, little is known about how often specific NPIs are being used and to what extent usage varies between countries. The aim of the current study was to investigate the prevalence of NPIs in older homecare recipients in six European countries.

**Methods:**

This is a cross-sectional study of older homecare recipients (65+) using baseline data from the longitudinal cohort study ‘Identifying best practices for care-dependent elderly by Benchmarking Costs and outcomes of community care’ (IBenC). The analyzed NPIs are based on the interRAI Home Care instrument, a comprehensive geriatric assessment instrument. The prevalence of 24 NPIs was analyzed in Belgium, Germany, Finland, Iceland, Italy and the Netherlands. NPIs from seven groups were considered: psychosocial interventions, physical activity, regular care interventions, special therapies, preventive measures, special aids and environmental interventions.

**Results:**

A total of 2884 homecare recipients were included. The mean age at baseline was 82.9 years and of all participants, 66.9% were female. The intervention with the highest prevalence in the study sample was ‘emergency assistance available’ (74%). Two other highly prevalent interventions were ‘physical activity’ (69%) and ‘home nurse’ (62%). Large differences between countries in the use of NPIs were observed and included, for example, ‘going outside’ (range 7–82%), ‘home health aids’ (range 12–93%), and ‘physician visit’ (range 24–94%).

**Conclusions:**

The use of NPIs varied considerably between homecare users in different European countries. It is important to better understand the barriers and facilitators of use of these potentially beneficial interventions in order to design successful uptake strategies.

**Supplementary Information:**

The online version contains supplementary material available at 10.1007/s41999-023-00868-w.

## Key summary points

**Aim:**

To investigate the prevalence of non-pharmacological interventions (NPIs) in older homecare recipients in six European countries.

**Findings:**

The prevalence of NPIs varied considerably between homecare users in different European countries. Interventions with a potential positive impact on health outcomes show a relatively low prevalence.

**Message:**

Further research into better implementation of potentially beneficial interventions in treatment guidelines might be needed, in order to optimize functioning and quality of life of older homecare recipients.

**Supplementary Information:**

The online version contains supplementary material available at 10.1007/s41999-023-00868-w.

## Introduction

In aging societies, the number of people living with complex chronic conditions is increasing rapidly. This increase will most likely lead to a rising number of people in need of long-term care, due to, for example, dependency in activities of daily living (ADLs) and/or instrumental activities of daily living (IADLs) [1].

Over the last decades, homecare has been marked as an effective alternative to long-term institutional care [2]. Homecare is not only a potentially cost-effective way of providing care, it is also the type of care preferred by care recipients [3]. This makes homecare one of the most important services to provide healthcare to older people with complex chronic conditions [2]. Studies have shown that there are still large differences in homecare policies and in the organization of homecare between different European countries, as well as in the availability of homecare services [4, 5].

Optimal care for older adults with chronic conditions includes both pharmacological and non-pharmacological interventions (NPIs) [6]. Some advantages of NPIs are that they can be combined with other NPIs as well as with pharmacological treatment without causing much interference and with minimal adverse side effects [7]. It is widely acknowledged that NPIs can be as effective as pharmacological therapies in the treatment of multiple chronic conditions [8, 9]. Traditionally, pharmacological interventions have received more attention through our disease-oriented healthcare system, even though NPIs are an important part of treatment according to clinical guidelines [10–12]. This may be due to multiple reasons, such as the lack of a common definition for NPIs, as well as methodological problems such as lack of a clear definition of interventions and how they are administered, and the lack of a theoretical model to explain the working mechanism of NPIs [13, 14]. In addition, there is also a perception that medication is easier to administer, and there may be a lack of reimbursement for NPIs. Moreover, funding sources for studying NPIs as compared to pharmacological interventions, are scarce [15].

While the emphasis is still on pharmacological treatment, the amount of research into the effectiveness of NPIs in the older population is growing [16–20]. However, insight into the use of NPIs in the wider homecare population is still lacking. Better insight into the use of NPIs may contribute to specific recommendations for NPI use and the application of treatment guidance in the older homecare population.

Therefore, the aim of this study was to describe the prevalence of NPIs in the older homecare population in six European countries.

## Methods

### Study design and data collection

For this cross-sectional analysis, we analyzed baseline data from the longitudinal cohort study ‘Identifying best practices for care-dependent elderly by Benchmarking Costs and outcomes of community care’ (IBenC). Data collection for this study was performed between January 2014 and August 2016, and the aim was to develop a benchmarking method for costs and quality of care of homecare organizations. The study rationale and protocol were described in detail elsewhere [21]. In short, a total of 2884 older adults receiving homecare (65 years and over) were enrolled from home healthcare organizations in Belgium, Finland, Germany, Iceland, Italy and the Netherlands. Participants were excluded if they (1) were terminally ill, (2) were expected to receive care only for a short term (< 6 months), (3) had planned institutionalization within 6 months, or 4) if they had no known informal caregiver or legal representative in the case of cognitive impairment [score on the Cognitive Performance Scale (CPS) ≥ 3] [19]. Detailed information was collected with the interRAI Home Care (HC) instrument. This instrument is a validated comprehensive geriatric assessment [22–25]. It consists of assessments of medical, psychological and social functional domains and service use, and assesses the needs of care-dependent older adults living in the community. The assessments were performed by trained nurses. Ethical approval was obtained before the start of the IBenC study from authorized medical ethical committees according to local regulations in each of the participating countries. Belgium (Flanders): Commissie Medische Ethiek van de Universitair Medische Ziekenhuizen Katholieke Universiteit Leuven, No. ML10265; Finland: Tutkimuseettinen työryhmä, No. THL/796/6.02.01/ 533/2014; Germany: Ethikkommission des Institut für Psychologie und Arbeitswissenschaft der Technische Universtität Berlin, No. GH_01_20131022; Iceland: Vísindasiðanefnd, No. 13–176-S1; Italy: Comitato Etico Università Cattolica del Sacro Cuore, No. 2365/14; The Netherlands: Medical Ethics Review Committee VU University Medical Center, No. 2013.333.

### Non-pharmacological interventions

The outcome measure was the use of non-pharmacological interventions (NPIs) and description of differences across analyzed countries. The prevalence of use of these interventions in the study population was analyzed using the interRAI HC instrument, which is an updated version of the interRAI MDS 2.0.

To determine which interventions could be defined as ‘non-pharmacological’, we held multiple consensus meetings with a multidisciplinary team of researchers and healthcare professionals from several European countries (the Netherlands, Poland and the Czech Republic). First, we discussed which descriptions of NPIs in the literature we agreed with [14, 26–28], since no clear definition of NPIs is available yet. Then, the teams from the different countries independently reported whether they considered the interRAI HC items NPIs or not (using the agreed definition/criteria for NPIs) and provided their argumentation. Subsequently, several consensus meetings were organized to discuss within the entire team which items were finally considered NPIs.

During these consensus meetings, we agreed that NPIs (1) involve interventions whose purpose is to improve, maintain or promote health or functioning; (2) aim to prevent, care or cure health problems, and have a measurable impact on health, quality of life, behavioral and/or socioeconomic markers; (3) are treatments which do not involve the use of drugs (but may be used in conjunction with pharmacological treatment); (4) are wide ranging in approach and encompass psychological, psychosocial, interpersonal, behavioral, emotional, exercise and environmental interventions; and (5) reflect a variety of approaches, and the creativity and commitment of many professional and lay carers of persons receiving care around the world.

We excluded diagnostic tools such as body sensors or pedometers, health campaigns focused on national level, variables that described a status more than something you could intervene with, such as walking performance or eating performance, and we excluded therapies we felt were pharmacological, such as oxygen therapy.

This eventually led to defining a list of 24 interventions we considered to be non-pharmacological among the available interRAI HC items. We assigned the interventions to seven groups by consensus: environmental interventions, physical activities, preventive measures, psychosocial interventions, regular care interventions, special aids and special therapies. The groups with corresponding NPIs can be found in Table 1.

**Table 1:** Defined groups and NPIs

**A. Psychosocial interventions**
Participation in social activities of long-standing interest last 7 days
Psychological therapy last 7 days
**B. Physical activity**
Exercise or physical activity in last 3 days
Went out of the house last 3 days
Physical therapy last 7 days
Occupational therapy last 7 days
**C. Regular care interventions**
Home nurse in last 7 days
Home health aids in last 7 days
Physician visit in last 90 days
Homemaking service in last 7 days
Meal care in last 7 days
**D. Special therapies**
Received wound care in last 3 days
Turning program in last 3 days
Received scheduled toileting program in last 3 days
Received palliative care in last 3 days
Speech therapy last 7 days
**E. Preventive measures**
Eye exam past year
Dental exam in past year
Hearing exam past year
Physical restraints
**F. Special aids**
Pads or briefs worn
Urinary collection device
**G. Environmental interventions**
Emergency assistance available
Re-engineered handicapped apartment

For analysis, all NPIs were transformed into dichotomous variables, stating whether a person received a certain NPI or not. Some NPIs that were measured in number of days given, were assigned as provided if the specified NPI was provided at least 1 day of the week. Extensive descriptions of the content of the interventions and of the period of measurement of the NPIs can be found in Supplementary Table 1.

### Demographics

Demographic characteristics included age, gender, living situation and country of residence. Living situation was defined as living alone or living with a person (e.g., partner, friend or other relative).

### Clinical characteristics

Functional status was measured using the Activities of Daily Living Hierarchy scale (ADLH), with scores ranging from 0 (no impairment) to 6 (total dependence). Higher scores indicate more ADL limitations, and a score of ≥ 3 indicates the need for extensive ADL support [21, 29]. To assess the self-performance on IADL (Instrumental Activities of Daily Living) tasks, the Instrumental Activities of Daily Living Capacity scale (IADLC) was used. This scale consists of eight IADL tasks that can be rated from independent (0) to complete dependence (6). The scores for each of the tasks were cumulated and range from 0 (independent) to 48 (total dependence) [23].

Cognitive impairment was evaluated using the Cognitive Performance Scale (CPS), with scores ranging from 0 (no cognitive impairment) to 6 (very severe cognitive impairment) [30]. Cut-off scores of ≥ 3 were used to indicate presence of moderate to severe cognitive impairment.

Presence of depressive symptoms was assessed using the 15-point Depression Rating Scale (DRS) [31]. A score of ≥ 3 indicates presence of clinically relevant depressive disorders.

The interRAI instrument has been linguistically validated with forward and back translations in many languages including Dutch, Finnish, German, Icelandic and Italian [32]. Also, interrater and test retest reliability was studied extensively in many countries and found to be very good [22, 32]. The cognition and depression scales are calculated from specific items from the interRAI instrument.

Other clinical characteristics taken into account included falls in the last 30 days, presence of incontinence, dyspnea, pain and fatigue, and self-reported health.

### Statistical analysis

Baseline characteristics were described using descriptive statistics such as mean and median. Descriptive statistics also provided an overview of the prevalence of NPIs in both the total sample and across the different European countries. We also counted the percentage of participants who received zero, one or more NPIs within each (previously defined) group of NPIs. All analyses were conducted with IBM SPSS statistics version 26 (IBM Corp., Armonk, NY, USA).

## Results

In total, 2884 participants from six European countries were included. The mean age at baseline was 82.9 years. Of all participants, 66.9% were female, and 57.7% were living alone. The percentage of participants needing extensive support in ADL was 41.4% in the total sample (range 8.4–82%). In the total sample, 16.6% suffered from moderate to severe cognitive impairment (range 1.8–37.1%). There was also a large variability in IADL capacity score between country samples (range 15.6–38.9). The participants’ characteristics are presented in Table 2.

**Table 2:** Baseline characteristics of the study population

Characteristics	Total	Belgium	Finland	Germany	Iceland	Italy	The Netherlands
	*n* = 2884	*n* = 525	*n* = 456	*n* = 493	*n* = 420	*n* = 499	*n* = 491
Age, years (median, IQR)	83.4 (78–88)	83.4 (77.5–87.5)	84.0 (78–88)	84.0 (79–89)	84.0 (79.5 – 89)	83.0 (76–87)	83.0 (78–87)
Female, *n* (%)	1930 (66.9)	352 (67.4)	313 (68.6)	351 (71.2)	292 (69.5)	286 (57.3)	336 (71.0)
Living alone, *n* (%)	1656 (57.7)	252 (48.9)	369 (80.9)	359 (72.8)	256 (61.0)	82 (16.4)	338 (69.4)
Extensive ADL support (ADLH ≥ 3), *n* (%)	1181 (41.4)	419 (82.0)	60 (13.2)	238 (48.4)	35 (8.3)	388 (80.3)	41 (8.4)
iADL capacity scale (0–48) (median, IQR)	29.0 (16–40)	35.0 (26–42)	27.0 (13–35)	27.0 (12–42)	24.0 (15–32)	44.0 (33–48)	13.0 (6–23)
Hours of informal care, last 3 days (median, IQR)	4.0 (1–15)	4.5 (1–16.5)	2.0 (1–3)	3.0 (1–9)	3.0 (1–9)	20.0 (9–30)	3.0 (1–7)
Moderate to severe cognitive impairment (CPS ≥ 3), *n* (%)	502 (16.6)	90 (17.9)	49 (10.7)	135 (27.4)	40 (9.5)	179 (37.1)	9 (1.8)
Depressive symptoms (DRS ≥ 3), *n* (%)	595 (20.9)	131 (25.6)	55 (12.1)	114 (23.2)	73 (17.4)	106 (21.9)	116 (23.6)
Pain present, *n* (%)	1437 (50.1)	258 (50.6)	278 (61.0)	214 (43.4)	252 (60.0)	217 (43.5)	218 (44.4)
Falls last 30 days, *n* (%)	366 (12.7)	87 (16.9)	50 (11.0)	32 (6.5)	44 (10.5)	85 (17.0)	68 (13.8)
Bladder incontinence, *n* (%)	1570 (54.7)	412 (79.5)	181 (39.8)	250 (50.8)	182 (43.3)	326 (65.6)	219 (44.7)
Dyspnea during activities or in rest, *n* (%)	898 (31.2)	185 (35.8)	85 (18.6)	97 (19.7)	178 (42.4)	146 (29.3)	207 (42.2)
Fatigue, moderate to severe, *n* (%)	987 (34.3)	202 (39.1)	62 (13.6)	139 (28.2)	230 (54.8)	257 (51.5)	97 (19.8)
Good self-reported health, *n* (%)	521 (24.1)	114 (23.2)	75 (17.9)	14 (60.9)	103 (24.9)	32 (9.7)	183 (37.9)

### Prevalence of non-pharmacological interventions

Supplementary Fig. 1 presents the ten most prevalent NPIs in the total homecare sample. The intervention with the highest prevalence was ‘emergency assistance available’ (74.1%), followed by ‘exercise or physical activity’ (68.9%) and ‘home nurse’ (61.9%).

Table 3 shows the prevalence of all NPIs in the total study sample, and within the six European countries. ‘Psychological therapy’ (0.6%) had quite a low prevalence. The proportion of older homecare clients receiving physical therapy was 14.6%, and the prevalence of occupational therapy was 5.6%. Physical restraints were present in 9.2% of the study sample.

**Table 3:** Prevalence of non-pharmacological interventions

Non-pharmacological interventions	Total sample	By country					
		Belgium	Finland	Germany	Iceland	Italy	The Netherlands
	*n* = 2884	*n* = 525	*n* = 456	*n* = 493	*n* = 420	*n* = 499	*N* = 491
Psychosocial interventions, *n* (%)							
Participation in social activities of long-standing interest last 7 days	943 (33.0)	150 (29.1)	132 (29.0)	289 (58.8)	81 (19.2)	41 (8.5)	250 (50.9)
Psychological therapy last 7 days	17 (0.6)	1 (0.2)	1 (0.2)	3 (0.6)	5 (1.2)	1 (0.2)	6 (1.2)
Physical activity, *n* (%)							
Exercise or physical activity in last 3 days	1970 (68.9)	363 (70.3)	441 (96.7)	305 (62.0)	354 (84.3)	82 (17.0)	425 (86.6)
Went out of the house last 3 days	1489 (52.1)	303 (58.9)	276 (60.5)	213 (43.3)	261 (62.1)	35 (7.2)	401 (81.7)
Physical therapy last 7 days	420 (14.6)	118 (22.5)	23 (5.0)	55 (11.2)	96 (22.9)	27 (5.4)	101 (20.6)
Occupational therapy last 7 days	161 (5.6)	43 (8.2)	1 (0.2)	110 (22.3)	3 (0.7)	0 (0.0)	4 (0.8)
Regular care interventions, *n* (%)							
Home nurse in last 7 days	1506 (61.9)	508 (100)	240 (52.6)	374 (75.9)	180 (42.9)	148 (29.7)	435 (88.6)
Home health aids in last 7 days	1738 (60.3)	233 (44.6)	424 (93.0)	420 (85.2)	313 (74.5)	60 (12.0)	288 (58.8)
Physician visit in last 90 days	1724 (60.3)	291 (58.3)	107 (23.5)	464 (94.1)	144 (34.3)	384 (77.0)	334 (68.0)
Homemaking service in last 7 days	1302 (45.2)	253 (48.2)	98 (21.5)	255 (51.7)	317 (75.8)	19 (3.8)	360 (73.5)
Meal care in last 7 days	682 (23.7)	113 (21.5)	245 (53.7)	92 (18.7)	112 (26.7)	14 (2.8)	106 (21.6)
Special therapies, *n* (%)							
Received wound care in last 3 days	321 (11.1)	81 (15.8)	22 (4.8)	45 (9.1)	20 (4.8)	114 (22.8)	39 (7.9)
Turning program in last 3 days	104 (3.6)	20 (3.9)	0 (0.0)	4 (0.8)	2 (0.5)	76 (15.2)	2 (0.4)
Received scheduled toileting program in last 3 days	90 (3.1)	22 (4.3)	10 (2.2)	13 (2.6)	7 (1.7)	34 (6.8)	4 (0.8)
Received palliative care in last 3 days	22 (0.8)	6 (1.2)	5 (1.1)	0 (0.0)	1 (0.2)	9 (1.8)	1 (0.2)
Speech therapy last 7 days	11 (0.4)	4 (0.8)	1 (0.2)	3 (0.6)	0 (0.0)	1 (0.2)	2 (0.4)
Preventive measures, *n* (%)							
Eye exam past year	1079 (37.7)	271 (54.2)	76 (16.7)	144 (29.2)	251 (59.8)	87 (17.4)	250 (50.9)
Dental exam in past year	747 (26.1)	141 (28.2)	61 (13.4)	169 (34.3)	136 (32.4)	42 (8.4)	198 (40.3)
Hearing exam past year	552 (19.3)	134 (26.7)	42 (9.2)	89 (18.1)	108 (25.7)	26 (5.2)	153 (31.2)
Physical restraints	216 (9.2)	N/A	2 (0.4)	63 (12.8)	5 (1.2)	138 (27.7)	8 (1.6)
Special aids, *n* (%)							
Pads or briefs worn	1682 (58.4)	373 (71.9)	222 (48.7)	311 (63.1)	201 (47.9)	334 (66.9)	241 (49.1)
Urinary collection device	198 (6.9)	27 (5.2)	6 (1.3)	28 (5.7)	19 (4.5)	90 (18.0)	28 (5.7)
Environmental interventions, *n* (%)							
Emergency assistance available	1906 (74.1)	438 (84.9)	410 (89.9)	143 (29.2)	384 (91.4)	127 (64.1)	404 (82.3)
Re-engineered handicapped apartment	903 (35.1)	91 (17.6)	65 (14.3)	306 (62.6)	220 (52.4)	33 (16.7)	188 (38.3)

### Prevalence of non-pharmacological interventions in different European countries

As can be seen from Supplementary Fig. 1 and Table 3, there were considerable differences in the prevalence of NPIs between the different European countries. NPIs with large differences between countries (i.e., large range in prevalence) were ‘participation in social activities’ (8.5–58.8%.), ‘exercise or physical activity’ (17.0–96.7%), ‘home health aids’ (12.0–93.0%), ‘eye exam’ (16.7–59.8%), and ‘emergency assistance available’ (29.2–91.4%).

### Prevalence of non-pharmacological interventions within the defined NPI groups

The prevalence of NPIs within different European countries was also analyzed with respect to previously defined NPI groups (Table 3 and Supplementary Fig. 1). Within the psychosocial interventions, ‘psychological therapy’ was rarely provided in all countries (0.2–1.2%). In the German sample, the prevalence of ‘occupational therapy’ was highest (22.3%) compared to the other countries (0.0–8.2%). Within the NPI group with regular interventions, there were large differences between the countries, with lowest prevalence in the Italian sample. In the German sample, there was a high prevalence of ‘physician visits’ (94.1%). In the group with special therapies, ‘wound care’ was most prevalent in the Belgian (15.8%) and the Italian samples (22.8%), ‘turning program’ and ‘toileting program’ were most prevalent in the Italian sample (15.2%; 6.8%). In the Finnish sample, nobody used a ‘turning program’ (0.0%). The group of NPIs with preventive measures included ‘physical restraints’, of which the prevalence was highest in the Italian sample (27.7%) and lowest in the Finnish sample (0.4%). Within the group with special aids a ‘urinary collection device’ was quite low prevalent, ranging from 1.3% in the Finnish sample to 18.0% in the Italian sample. The prevalence of ‘emergency assistance available’ within the environmental NPI group was high (74.1%), but ranges from 29.2% in the German sample to 91.4% in the Icelandic sample.

### Number of interventions within the different groups of NPIs

In Table 4, the proportion of people receiving a certain number of interventions is shown across the different NPI groups. People in the Dutch sample received the highest number of physical activity interventions (15.9% receiving three or more interventions), and the highest number of preventive measure interventions (30.3% with two interventions and 8.7% with three or more interventions). The number of physical activity interventions was lowest in the Italian sample, with 79.0% receiving no interventions. In the Finnish sample, the number of preventive measures was the lowest with 71.3% of the study sample receiving no interventions from this group. The number of environmental interventions was highest in Iceland, with 48.7% of the sample receiving two interventions. Within the regular care group, the German sample showed 46.6% of the people receiving four or more interventions. Within the special aids group and the special therapies group, the differences between countries were smaller.

**Table 4:** Number of non-pharmacological interventions received by intervention category

Number of interventions	Total sample	By country					
		Belgium	Finland	Germany	Iceland	Italy	The Netherlands
	*n* = 2884	*n* = 525	*n* = 456	*n* = 493	*n* = 420	*n* = 499	*N* = 491
Psychosocial interventions, %							
0	67.1	71.4	70.8	41.5	79.8	91.8	48.8
1	32.6	28.4	29.2	57.9	20.0	8.0	50.4
2	0.3	0.2	0	0.6	0.2	0.2	0.8
Physical activity, %							
0	22.1	13.7	1.3	19.4	9.7	79.0	6.1
1	26.0	29.1	38.2	35.6	24.9	14.4	14.6
2	42.3	45.3	57.5	33.4	51.3	5.4	63.4
≥ 3	9.6	11.8	3.1	11.5	14.0	1.2	15.9
Regular care interventions, %							
0	4.3	1.1	0.4	0.2	1.2	15.2	6.9
1	19.2	11.4	15.4	2.4	14.0	52.1	18.7
2	29.8	31.4	39.3	20.9	36.6	26.9	25.6
3	27.4	34.9	31.8	30.0	30.6	4.0	33.9
≥ 4	19.3	21.2	13.1	46.6	17.6	1.8	14.8
Special therapies, %							
0	84.3	78.9	92.3	88.3	93.8	64.1	90.9
1	13.0	17.3	7.0	10.5	5.5	26.9	8.5
≥ 2	2.7	3.8	0.7	1.2	0.7	9.0	0.6
Preventive measures, %							
0	43.9	38.1	71.3	49.4	25.7	53.9	24.6
1	30.1	28.4	19.5	21.9	38.7	36.1	36.4
2	18.8	25.0	7.5	15.6	26.8	7.8	30.3
≥ 3	7.3	8.6	1.8	13.1	8.8	2.2	8.7
Special aids, %							
0	39.5	28.2	50.7	35.0	49.2	29.5	47.4
1	56.0	67.4	48.7	61.3	49.4	56.1	50.6
2	4.6	4.4	0.7	3.6	1.4	14.4	2.0
Environmental interventions, %							
0	25.6	15.0	8.8	33.6	5.2	73.3	13.2
1	51.6	69.1	78.3	41.9	46.1	21.2	53.3
2	22.9	15.8	12.9	24.5	48.7	5.4	33.5

## Discussion

This study described the prevalence of the provision or use of NPIs in homecare recipients across six European countries, as well as the number of homecare recipients receiving zero, one or more interventions  within the different NPI groups. The prevalence of NPIs varied substantially, both within and across countries. Low prevalent interventions concerned ‘participation in social activities’, ‘psychological therapy’, ‘physical therapy’ and ‘occupational therapy’. The use of physical restraints was relatively high in the Italian and German samples. The fewest number of interventions per person occurred in the Italian sample.

In this homecare European study sample, the prevalence of certain interventions, such as ‘social participation’, ‘physical therapy’ and ‘occupational therapy’ was low. This is striking, because these are all interventions that may have a positive effect on different health outcomes. Physical and occupational therapy positively impact reducing the recurrence of falls and subsequent morbidity and mortality [33], and occupational therapy improves mobility, social participation and ADL functioning [34], and also daily functioning, particularly in people with dementia [35]. Moreover, physical activity improves ADL functioning in older people [36]. Social participation has a positive effect on cognitive, emotional and physical functioning in older people [37].

The relatively high prevalence of use of physical restraints in Italy and Germany may have a negative effect on health outcomes such as emotional wellbeing and physical functioning while evidence for their effectiveness on for instance fall prevention is lacking. Use of physical restraints has been associated with increased risk of falls, a decline in physical functioning, pain, mental health problems, a higher risk of pressure ulcers, incontinence and injuries [38–41].

When interpreting our results, it is important to consider that the organization and availability of care as well as the characteristics of the samples differ across European countries. Previous research has shown that the amount of formal care is higher in the Netherlands, Belgium and Scandinavian countries compared to Mediterranean countries where people receive more informal care [42]. In this study, the amount of informal care given in the Italian sample is also higher than in the other countries [21]. The ratio of formal and informal care varied substantially [43]. Future research may explore whether and how this may have impacted the uptake of various types of NPIs.

There were also sample differences between the different countries that might in part explain the differences between the use of NPIs. The Italian and Belgian samples had the highest numbers of people needing extensive ADL support (80.3 and 82.0%). Also, the number of people with moderate to severe cognitive impairment was highest in Italy, with 37.1%. This might partly explain the high number of physical restraints compared to the other countries, since one of the major risk factors for physical restraints use is cognitive impairment or dementia [40, 41]. It may also explain the low number of physical activity NPIs, because physical restraints are also more prevalent in people with impaired ADL or higher care dependency [40, 41]. Apart from this, there were also different eligibility criteria for homecare in different countries. In Italy, the indication for receiving homecare is aimed more at ADL-dependent and cognitively impaired persons. In the participating Italian region, the indication for receiving homecare was aimed at relatively severe ADL-dependence and cognitive impairment. As there is very little nursing home capacity in the Italian region (Umbria) included in this study, people are cared for at home much longer compared to the other countries. At the same time, homecare capacity is rather limited as well leaving much of the daily care with informal carers. Informal carers may be less aware of supportive interventions. Likely, this context drives the limited uptake of NPIs in Italy despite serving very care-dependent clients. In the Netherlands, Finland and Iceland, less impaired people are also eligible for homecare service provision. The differences between homecare eligibility and levels of dependency in these countries have been described previously [44].

Our study has some strengths and limitations. The strengths of our study are that data were collected using the standardized and validated interRAI HC instrument, available in various country-specific editions, in which assessors are specifically trained to ensure appropriate research and clinical use of this instrument. An advantage was also that all NPIs were explicitly and uniformly defined before analyses based on consensus of experts from several countries on the definition and selection of NPIs from the interRAI HC tool and classification of these NPIs into specific groups.

One of the limitations of the study is that the data are from 2014 to 2016, with no access to data from during or after the COVID-19 pandemic. However, comprehensive data on NPIs from multiple countries are relatively scarce. Our dataset is one of the few that allow such detailed international comparisons. Another limitation is that in some countries the sampling procedure might have contributed to selection bias. For example, in the Dutch study sample, one of the main reasons for refusal to participate in two of the three study sites was cognitive impairment, likely causing an underrepresentation of older persons with cognitive impairment [21]. The Belgian study sample was recruited from a nursing homecare organization, possibly causing the higher levels of ADL-dependency and cognitive impairment. This would also explain why all participants had a home nurse. Also, one of the inclusion criteria for this study was the expectation that participants would receive care for more than 6 months, so the results relate to people who received “long-term” homecare. This may influence the generalizability of the present study. People with acute or subacute conditions might require different NPIs such as physical or occupational therapy, psychological or speech therapy, pads or briefs worn or urinary collection device. Terminally ill persons were also excluded from the study population, so people receiving palliative care in this study sample were underrepresented. Another limitation is the fact that countries differ in their spending on and eligibility criteria for homecare, which may cause populations eligible for provision of homecare services to differ from country to country. Also, specific customs in provision of some NPIs may play a significant role in their higher provision, but these findings are also part of the results of our study.

Concerning the NPIs, we were dependent on the NPIs listed in the interRAI HC instrument. As a result, the list of NPIs might be incomplete. For example, specific nutritional interventions were not captured with this instrument. This can be seen as a limitation to the current study. Also, details about intensity and performance of NPIs were largely unknown. The assessment using the HC instrument is a snapshot, so we do not know if more NPIs are deployed during the treatment trajectory.

Also, we did not consider whether the study population did or did not have an indication for a particular NPI, which might cause an over- or underestimation of their prevalence.

There were large differences in the prevalence of different NPIs across European countries. Some interventions with positive effects were hardly used, such as ‘participation in social activities’, ‘psychological therapy’, ‘physical therapy’ and ‘occupational therapy’. Interventions such as ‘physical restraints’ were highly prevalent in the Italian and the German study samples but might have a negative impact on health outcomes such as emotional wellbeing.

Remarkably, several interventions with proven benefit were deployed only on a modest scale. The allocation criteria for NPIs differ substantially across countries. Nevertheless, it seems important to better understand the impeding and facilitating factors in the use of these potentially beneficial interventions in order to design successful uptake strategies. Also, further research into better implementation of these interventions in treatment guidelines might be needed. In most countries, better uptake of NPIs seems to be a window of opportunity to improve functioning and quality of life of homecare recipients.

### Supplementary Information

Below is the link to the electronic supplementary material.

Supplementary file1 (PDF 1348 KB)

## Declarations

### Availability of data and materials

The datasets used and/or analyzed during the current study are available from the corresponding author on reasonable request and with permission from the IBenC consortium.
