# Supplementary material for: The prevalence of non-pharmacological interventions in older homecare recipients: an overview from six European countries
Source: Eur Geriatr Med. 2023 Oct 4;15(1):243–52. doi: 10.1007/s41999-023-00868-w (PMC10876758; doi:10.1007/s41999-023-00868-w)

Supplementary Information

Supplementary Table 1. List of selected non-pharmacological interventions per NPI group

Intervention	Description according to interRAI guidelines
Psychosocial interventions	
Participation in social activities	**Participation in social activities of long-standing interest** — The person engaged in social activities that have been of long-standing interest to him or her. The activities may be quite varied and should be counted as long as they involve interaction with at least one other person. Examples include attending meetings of informal clubs or religious services, playing bridge or bingo, volunteering at the local clothing bank, or gossiping with the neighbors on their front porches in the evening.
Psychological therapy	**Psychological therapy** — Therapy given by a licensed mental health professional, such as a psychiatrist, psychologist, psychiatric nurse, or psychiatric social worker.
Physical activity	
Physical therapy	**Physical therapy** — Therapy services that are provided or directly supervised by a qualified physical therapist. A qualified physical therapy assistant may provide therapy, but may not supervise others giving therapy.
Occupational therapy	**Occupational therapy** — Therapy services that are provided or directly supervised by a qualified occupational therapist. A qualified occupational therapy assistant may provide therapy, but may not supervise others giving therapy.
Exercise or physical activity	**Exercise or physical activity —** Any exercise that involves at least moderate physical activity, such as walking outdoors, swimming, yoga, class, exercise using exercise machines.
Went out of the house	**Went out of the house or building —** This means the person went outdoors, no matter how short the period of time he or she spent outdoors. This could mean going into the yard, standing on an open porch, or walking down the street.
Regular care interventions	

Home health aides	**Home health aide —** Aides who traditionally provide "hands-on" ADL support and simple monitoring (such as taking blood pressure).
Home nurse	**Home nurse —** Licensed or registered nurses who traditionally provide assessment and complex or invasive interventions (skilled treatments), education, and referral.
Physician visit	**Physician visit in last 90 days** - A visit to a medical provider's office or clinic by the person, or a medical provider's visit to the person's home. This item includes a very broad spectrum of medical providers and specialists—for example, MDs or osteopaths, who may be either the primary physician or consultant(s); authorized physician's assistants; or nurse-practitioners.
Homemaking service	**Homemaking services** — Services that traditionally include IADL support, usually in the form of housekeeping services, shopping, and meal preparation
Meal care	**Meals —** Prepared meals that are delivered to the person for immediate or later consumption (for example, meals-on-wheels).
Special therapies	
Wound care	**Wound care** — Includes the application of bandages (for example, dry gauze dressings, dressings moistened with saline or other solutions, transparent dressings, hydrogel dressings, or dressings with hydrocolloid or hydroactive particles); wound irrigation; the application of ointments and topical medications to treat skin conditions (for example, cortisone, antifungal preparations, or chemotherapeutic agents); debridement (chemical or surgical) to remove dirt or dead tissue from a wound; and suture removal.
Toileting program	**Scheduled toileting program** — The person is taken to the toilet room, given a urinal, or reminded to go to the toilet on a regular and ongoing basis. In the home, this may be done by

	family members or paid help. Includes any habit training or prompted voiding program
Turning program	**Turning/repositioning program —** The person is periodically turned from side to side and onto his or her back while in bed. Once the person has been turned to the new side, staff ensure that the head, torso, and limbs are positioned to minimize pain, promote function, and minimize pressure on bony prominences.
Palliative care	**Palliative care program —** A formal program in which care is focused on the relief of pain and other uncomfortable symptoms (such as dyspnea). Persons receiving palliative care generally have end-stage disease, but may or may not have a prognosis of 6 months or less to live (i.e., the person may live for many months or years).
Speech therapy	**Speech-language pathology and audiology services** — Services provided by a qualified speech-language pathologist. Services may involve assessment of swallowing ability or hearing ability, swallowing therapy, speech therapy, communication therapy, providing hearing appliances, and education.
Preventive measures	
Dental exam	**Dental exam in last year** — The person underwent a dental examination by a dentist, oral surgeon, or dental hygienist during the past year.
Eye exam	**Eye exam in last year** — The person underwent an eye examination by an ophthalmologist, optometrist, physician, nurse, or other clinician within the past year.
Hearing exam	**Hearing exam in last 2 years** — The person underwent a hearing examination by an audiologist or other clinician within the past 2 years.
Physical restraints	For example, the person's limbs were restrained; the person used bed rails; or the person was restrained to the chair when sitting.
Special aids	

Urinary collection device	**External (condom) catheter** — A urinary collection device worn over the penis.<br><br>**Indwelling catheter** — A catheter that is maintained within the bladder for the purpose of continuous drainage of urine. Includes catheters inserted through the urethra or by suprapubic incision.<br><br>**Cystostomy** — An opening to the bladder made by a surgical incision and covered by a urinary collection appliance (urostomy bag).<br><br>**Nephrostomy** — A tube, stent, or catheter that is used to provide urinary drainage when a ureter is obstructed. In some cases the catheter drains urine out of a person's body into a drainage bag. In other cases, the catheter drains urine directly into the bladder.<br><br>**Ureterostomy** — A surgical urinary diversion, where the ureter(s) is detached from the bladder and brought to the surface of the abdomen, with a urinary collection device placed over it.
Pads or briefs worn	Any type of absorbent, disposable or reusable undergarment or item, whether worn by the person (for example, a diaper or adult brief) or placed on the bed or chair for protection from incontinence. Does not include the routine use of pads on beds when the person is never or rarely incontinent.
Environmental interventions	
Re-engineered handicapped apartment	Lives in apartment or house re-engineered accessible for persons with disabilities.
Emergency assistance	**Availability of emergency assistance** — For example, telephone, alarm response system. The person indicates that he or she has access to emergency assistance. This could be by means of a telephone, or speed dialing option on the telephone, or an emergency response system.

Supplementary Figure 1. Top 10 most prevalent non-pharmacological interventions (NPIs) compared between the 6 European countries, ordered from high to low prevalence in the total sample

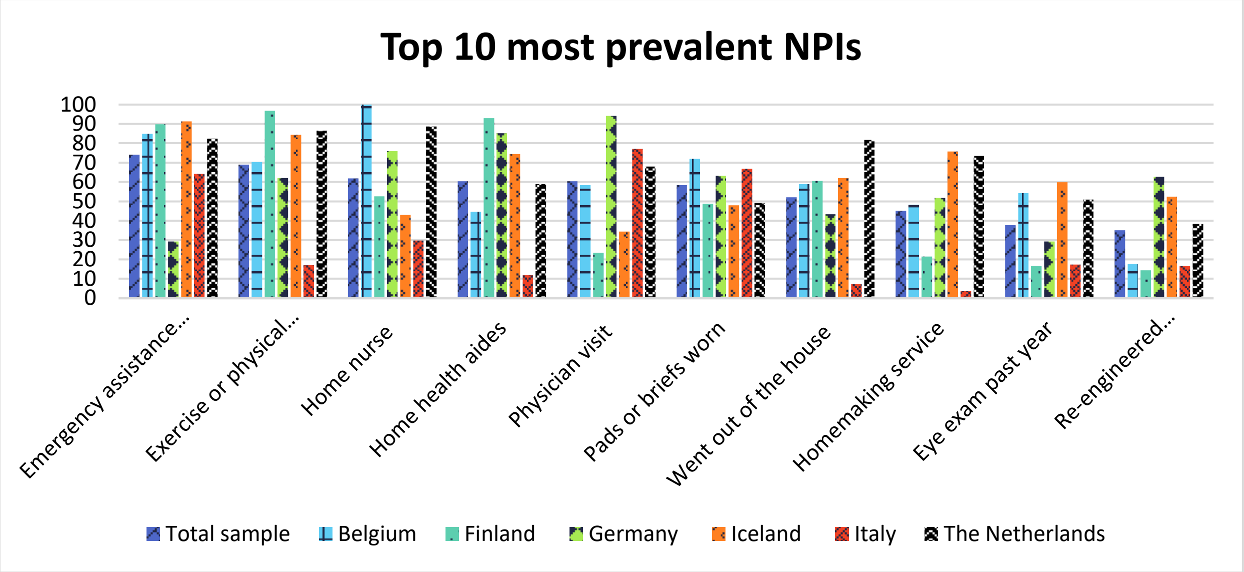

Supplementary Figure 2. Prevalence of NPIs within the different groups in six European countries

2A. Prevalence of NPIs within the psychosocial interventions group in different countries

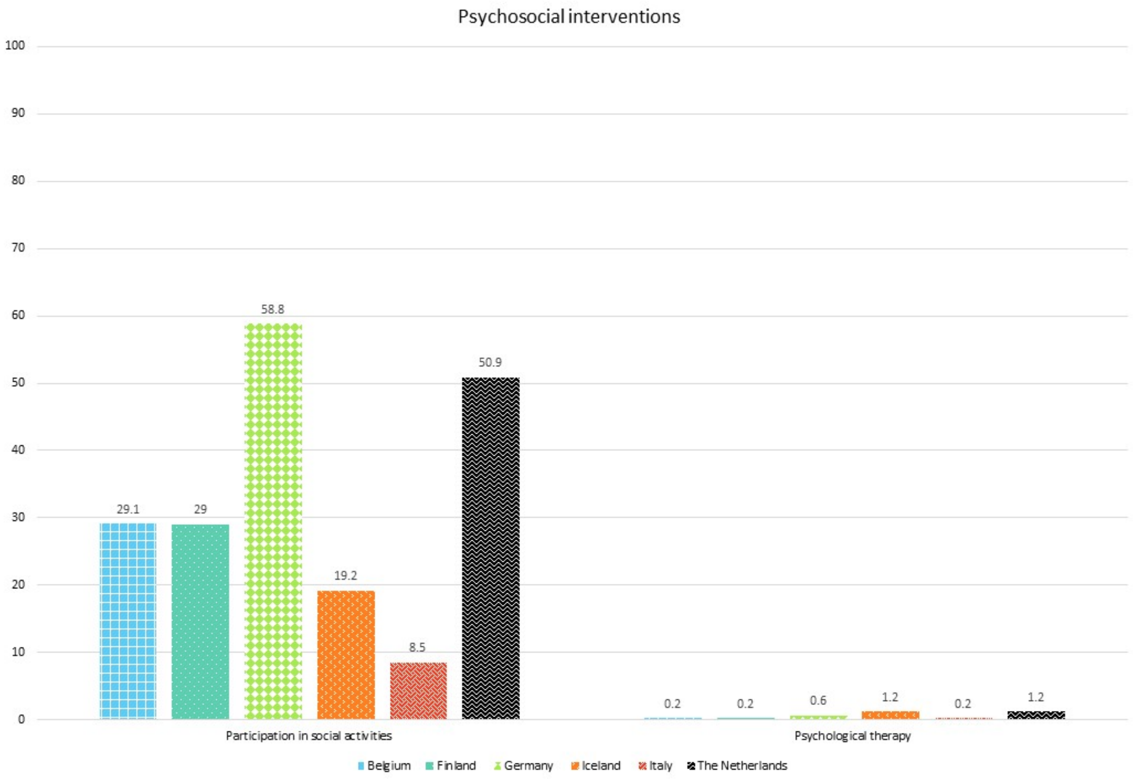

2B. Prevalence of NPIs within the physical activity group in different countries

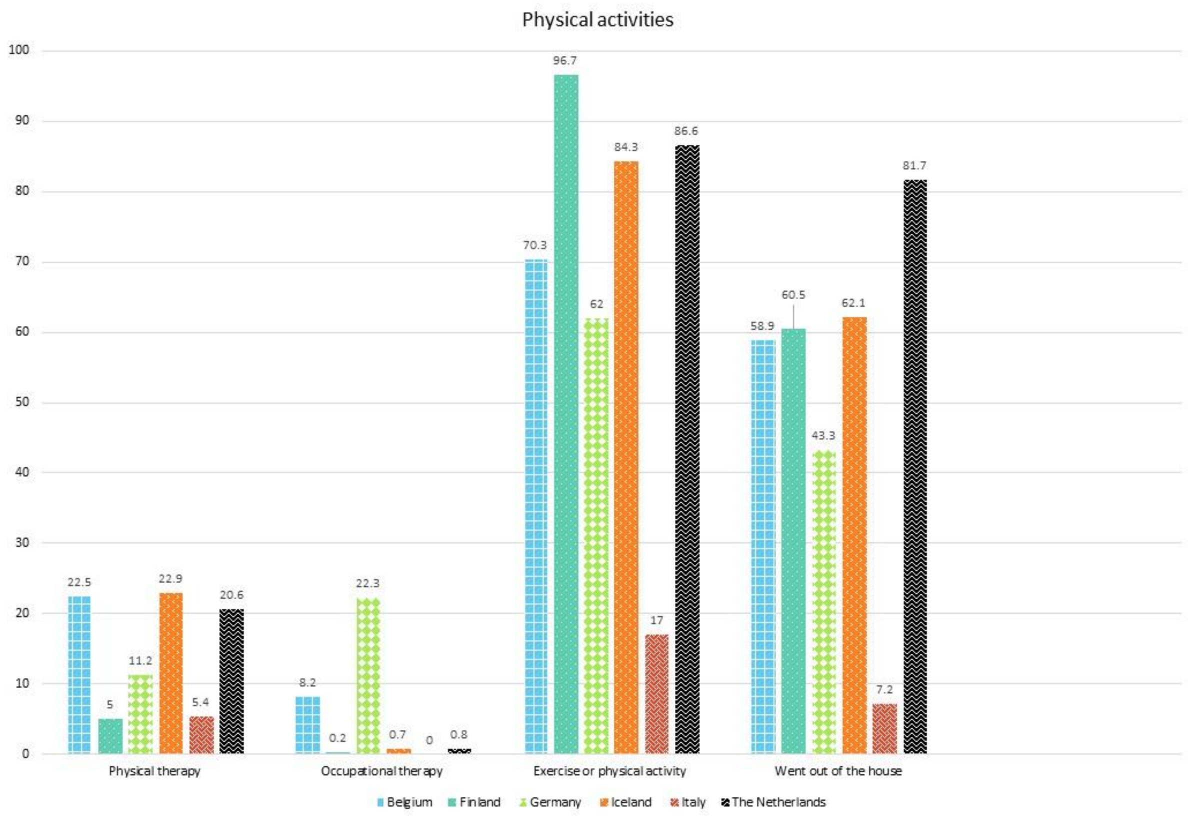

2C. Prevalence of NPIs within the regular care group in different countries

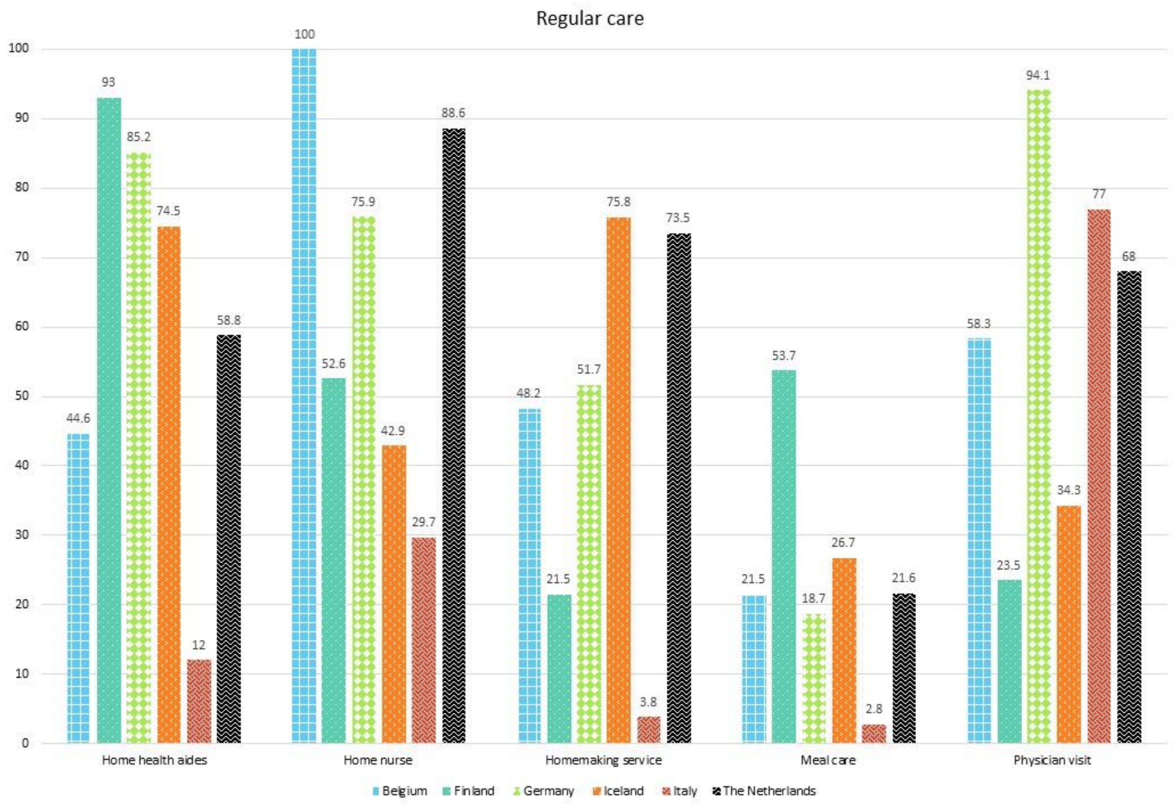

2D. Prevalence of NPIs within the special therapies group in different countries

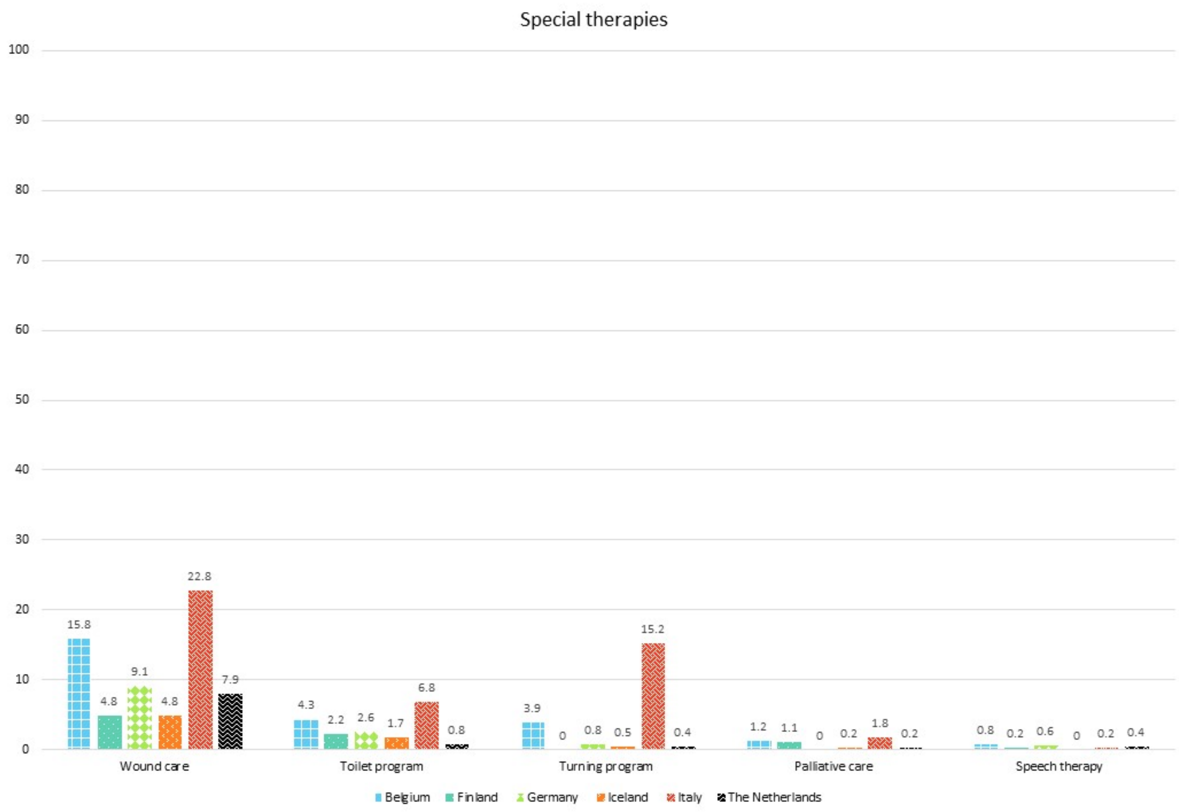

2E. Prevalence of NPIs within the preventive measures group in different countries

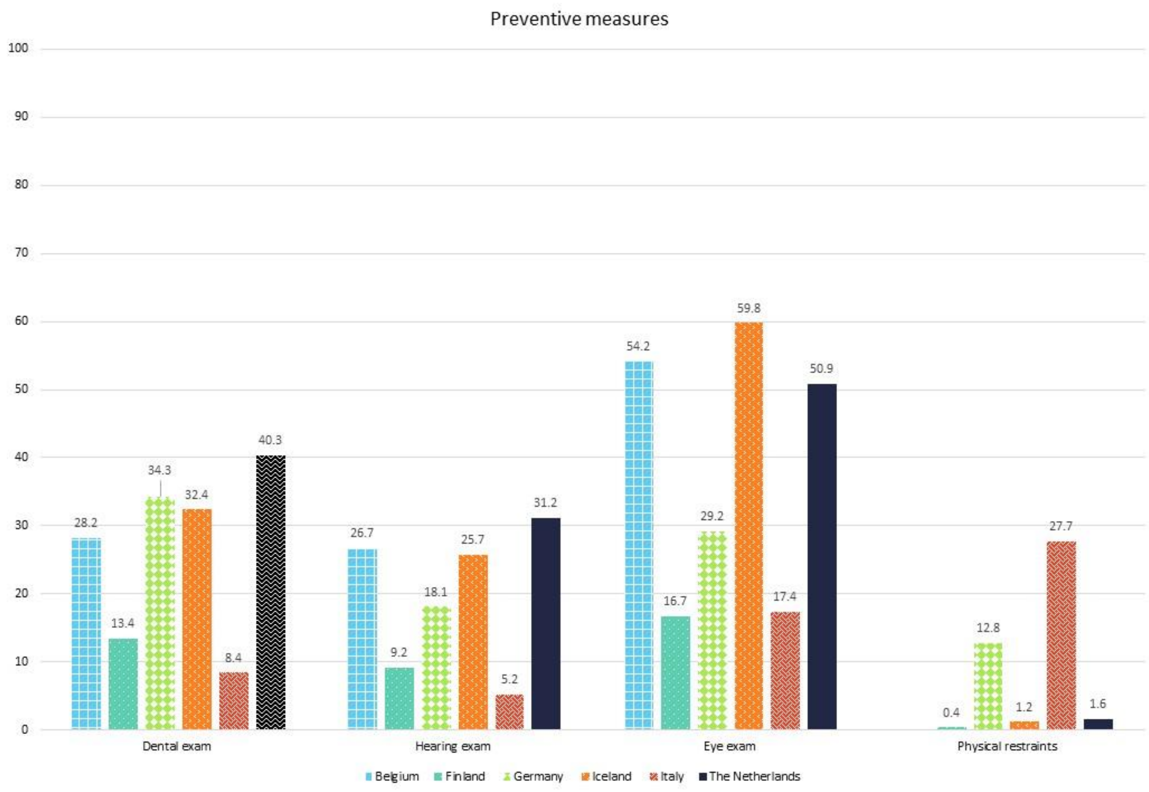

2F. Prevalence of NPIs within the special aids group in different countries

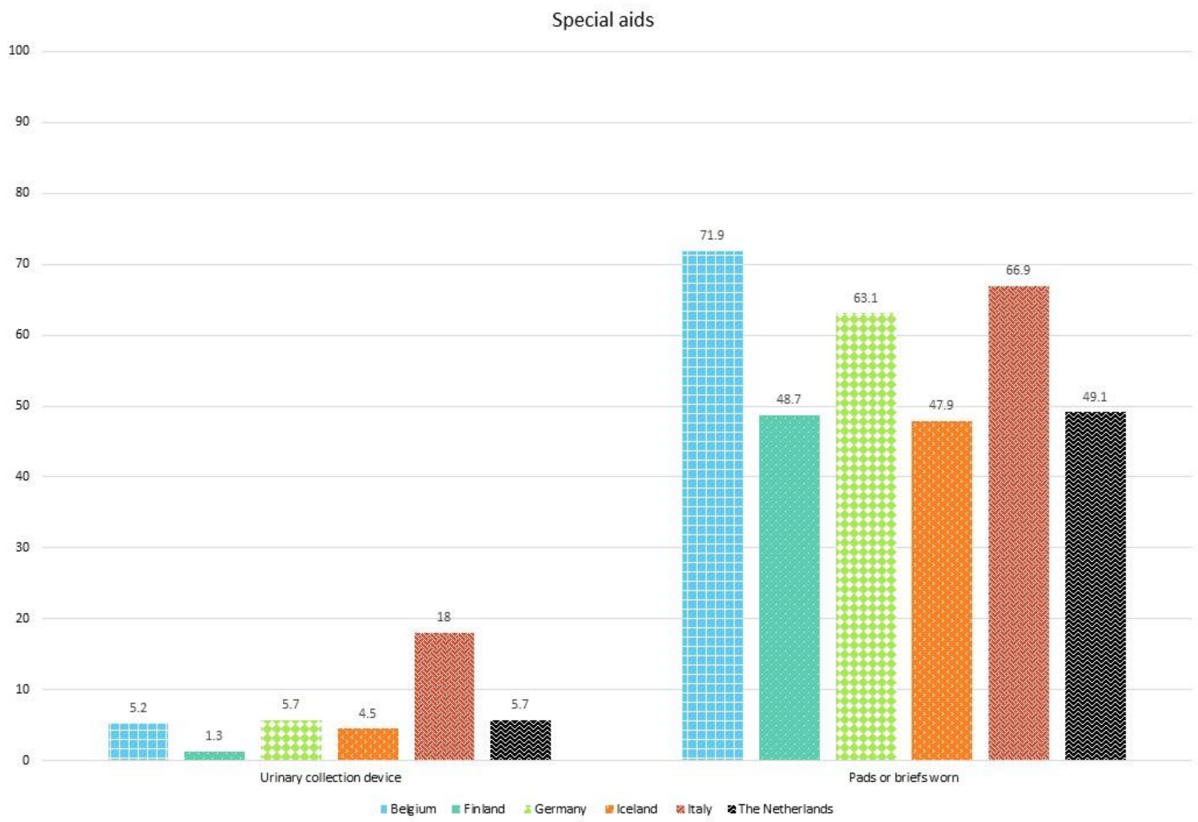

2G. Prevalence of NPIs within the environmental interventions group in different countries

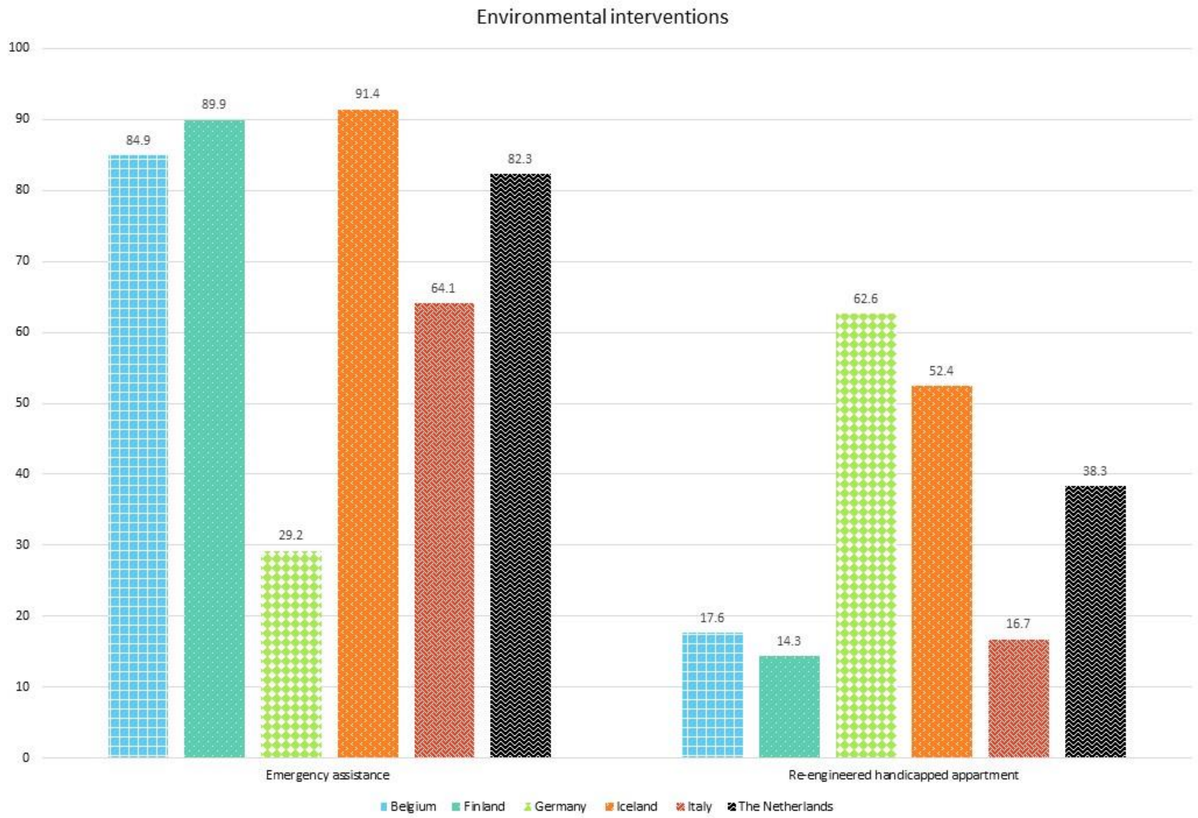

Supplement: Supplementary file 1 — Supplementary file1 (PDF 1348 KB) [file 41999_2023_868_MOESM1_ESM.pdf]
